# Frailty among a group of the elderly people in Morocco: prevalence and associated factors

**DOI:** 10.11604/pamj.2024.49.123.44645

**Published:** 2024-12-17

**Authors:** Zineb Bouzaher, Noureddine El Khoudri

**Affiliations:** 1 Laboratory of Health Sciences and Technologies, Higher Institute of Health Sciences, Hassan First University, Settat, Morocco

**Keywords:** Elderly, Fried frailty phenotype, frailty, prevalence, Morocco

## Abstract

**Introduction:**

frailty is a common syndrome among elderly people, but has not been fully studied in Morocco. We aimed to identify frailty in a group of non-institutionalized elderly people aged 60 and over in Morocco and to estimate its prevalence. Additionally, we sought to identify the factors associated with frailty in this specific population.

**Methods:**

this multicentric cross-sectional study involved 883 participants aged 60 years and over, distributed across three regions of Morocco, conducted between February and July 2023. To assess frailty, we relied on Fried's phenotype criteria, including involuntary weight loss, exhaustion, low physical activity, slow walking speed, and muscle weakness. Additionally, we examined participants' sociodemographic characteristics, health profiles, as well as their ability to perform activities of daily living and instrumental activities of daily living.

**Results:**

according to the Fried phenotype criteria, the prevalence of frailty was 35.2%, pre-frailty was 35.8%, and 29% of the elderly were robust. Frailty and pre-frailty were higher in women than in men. Multivariate analyses showed that frailty was associated with age, place of residence, subjective health, as well as activities of daily living and instrumental activities of daily living.

**Conclusion:**

the high prevalence of frailty in this study underscores the urgent need to develop targeted interventions aimed at preventing or delaying its progression among elderly people. By doing so, we can improve their quality of life while also alleviating the strain on healthcare systems.

## Introduction

At the global level, population aging is one of the most significant demographic phenomena. By 2050, one in six people worldwide will be over 65 years old (16%), compared to one in eleven in 2019 (9%) [1].

Morocco is no exception to the global phenomenon of aging [2]. It has undergone a demographic transition characterized by an increase in life expectancy and a decrease in fertility rates [3,4]. According to projections by the High Commission for Planning (HCP), by 2030, the proportion of elderly people aged 60 and over is expected to reach 15.4%, which translates to nearly 6 million elderly people [4]. The latest General Census of Population and Housing (RGPH) revealed that there were approximately 3.2 million elderly aged 60 and over in 2014 [5]. Therefore, this figure is expected to nearly double by 2030 [5]. This demographic trend highlights the growing importance of addressing the issues related to an aging population in Morocco.

Elderly people constitute a diverse population, characterized by significant interindividual variability and specific health needs. Chronological age alone is insufficient to define old age, as it does not directly reflect the level of dependency. Frailty, as a precursor state to functional dependency, holds crucial importance due to its reversibility, thereby providing an opportunity for early intervention to enhance the health and well-being of the elderly [6]. Frailty can be defined as a state of instability associated with an increased risk of functional autonomy loss. According to Brown (1995), frailty is characterized by a decline in the ability to perform essential physical and social activities of daily life, with insufficient reserves to cope with potential stressors or insults [7]. The frailty syndrome serves as a risk marker for mortality and adverse events, including disabilities, falls, hospitalization, and institutionalization [8].

Different theoretical models address frailty in elderly people, each offering a unique perspective depending on the explored domain, ranging from physiological to socio-environmental dynamics. Regardless of the model defining the frailty syndrome, its consequences are manifold, including overall mortality, functional decline, falls, hospitalizations, as well as changes in living arrangements such as institutionalization [8]. The prevalence of frailty varies widely in the literature, influenced by the studied population and the measurement tools used.

In this study, we aimed to identify frailty in a group of non-institutionalized elderly people aged 60 and over in Morocco and to estimate its prevalence. Additionally, we sought to identify the factors associated with frailty in this specific population, using the Fried frailty phenotype as a reference. To our knowledge, no study on frailty among non-institutionalized elderly people has been published in Morocco yet, highlighting the importance of this research to fill this scientific gap and better understand the challenges related to frailty in our national context.

## Methods

**Type, location, and design of the study:** we conducted a multicenter, cross-sectional, descriptive, and analytical study among elderly people aged 60 and over attending urban and rural health centers in three regions of Morocco: Casablanca-Settat, Béni Mellal-Khénifra, and Marrakech-Safi, between February and July 2023. The study was carried out across 12 primary healthcare centers, both rural and urban, within these regions.

**Study population:** the participants, all elderly aged 60 and over, were recruited randomly from urban and rural health centers during their visits. Each participant gave oral consent to take part in the study. The study specifically included non-institutionalized elderly individuals. Participants were randomly approached and included in the study if they met the predefined inclusion criteria.

**Study size:** our sample consisted of 883 individuals. The sample size was determined based on available resources, participant accessibility, and the time allocated for data collection.

**Data collection:** data were collected using a structured questionnaire consisting of four distinct sections. The questionnaire, in paper format, was administered by trained members of the research team at the health centers, who conducted face-to-face interviews with the participants. Additionally, we measured participants' height and weight using a tape measure and an electronic scale, respectively, and assessed their walking speed. Muscle strength was measured with a Jamar dynamometer. The questionnaire comprised the following sections: 1) Sociodemographic characteristics: sex, age, family status, level of education, and living arrangements; 2) health-related characteristics: the presence of chronic diseases, medical follow-up, subjective health, and Body Mass Index (BMI); 3) assessment of Activities of Daily Living (ADL) and Instrumental Activities of Daily Living (IADL); 4) frailty assessment according to Fried Frailty Phenotype (FFP).

Frailty assessment was conducted according to the FFP developed by Fried *et al*. comprising five distinct criteria [9]. 1) Unintentional weight loss: This condition was defined as a decrease in body weight of ≥ 4.5 kg or 5% over 1 year, without the use of special methods such as diets or medications [9]; 2) exhaustion: this criterion was considered positive when frequent fatigue was reported in response to the question “do you often feel tired?” [10]; 3) low physical activity: assessment of this criterion was based on the Physical Activity Scale for the Elderly (PASE) tool, categorizing participants as active if they engaged in light daily activities or weekly sports activities, and as inactive if they did not [11]; 4) slowed walking speed: to measure mobility, Fried's model relies on measuring walking speed. Slowness is considered present if the individual cannot cover a distance of 4 m, at their usual pace, within a specific time [12]. The time required to perform this test is adjusted based on the participant's sex and height as follows: men: ≤ 173 cm, frail if time ≥ 6 sec, >173 cm, frail if time ≥ 5 sec; women: ≤ 159 cm, frail if time ≥ 6 sec, > 159 cm, frail if time ≥ 5 sec; 5) muscle weakness: grip strength was measured using a Jamar dynamometer, with a maximum value lower than 26 kg in men or 18 kg in women was defined as muscle weakness [10]. According to the FFP, among the five criteria mentioned, individuals with none were considered robust, those with 1 to 2 criteria were pre-frail, and those with 3 or more criteria were considered frail.

**Data analysis:** the collected data were entered and analyzed using IBM SPSS (Statistical Package for the Social Sciences) software version 25.0. Initially, descriptive analysis was conducted to describe the characteristics of the participants. Subsequently, we used the chi-square test to study the association between frailty among groups of older adults and qualitative or categorical variables. To study the association between frailty and quantitative variables, the ANOVA test was employed. Additionally, binary logistic regression analysis was applied to explore the factors associated with frailty. Statistical significance was determined at a 5% threshold.

**Ethical considerations:** the study was carried out after obtaining the necessary authorizations from the health authorities.

## Results

**Socio-demographic and health profile of the elderly people studied:** the study conducted between February and July 2023, in three regions of Morocco, included a total of 883 participants aged 60 and over. The mean age of the participants was 68.8 ± 7.78 years, with a significant difference between sexes, where men had an average age of 69.90 ± 8.145 years, and women had an average age of 68.08 ± 7.451 years, p <0.01.

The majority of participants resided in rural areas, accounting for 72.6% of the total, with 75.1% being men and 70.9% being women. Furthermore, 80.4% of participants had no formal education, among whom 86.7% were women and 71.1% were men. Regarding health status, a large proportion of participants (80.7%) suffered from chronic diseases, with a higher prevalence among women (82.9%) than among men (77.6%). Hypertension (HTA) and diabetes were the most commonly observed pathologies, affecting 52.7% and 34.9% of participants, respectively (Table 1).

**Table 1:** comparison of general conditions between female and male

Variables		Total/mean	Male	Female	P
			(Number/percentage)	(Number/percentage)	
**Age, years, mean (SD)**		**68.8± 7.78**	**69.90 ±8.145**	**68.08 ±7.451**	**<0.001**
Age class	60-69	522	189 (52.9%)	333 (63.3%)	0.001
	70-79	247	105 (29.4%)	142 (27%)	
	≥80	114	63 (17.6%)	51 (9.7%)	
Residence area	Urban	242	89 (24.9%)	153 (29.1%)	0.174
	Rural	641	268 (75.1)	373 (70.9%)	
Marital status	Single	20	4 (1.1%)	16 (3%)	<0.001
	Married	509	263 (73.7%)	246 (46.8%)	
	Divorced	42	9 (2.5%)	33 (6.3%)	
	Widowed	312	81 (22.7%)	231 (43.9%)	
Education level	No schooling	710	254 (71.1%)	456 (86.7%)	<0.001
	Primary	113	67 (18.8%)	46 (8.7%)	
	Secondary	60	36 (10.1%)	24 (4.6%)	
Living arrangement	Alone	137	39 (10.9%)	98 (18.6%)	0.002
	With family	746	318 (89.1%)	428 (81.4%)	
Subjective health	Good	232	117 (32.8%)	115 (21.9%)	<0.001
	Average	228	109 (30.5%)	119 (22.6%)	
	Bad	349	102 (28.6%)	247 (47.0%)	
	Very bad	74	29 (8.1%)	45 (8.6%)	
Medical follow-up	Yes	464	176 (63.5%)	288 (66.1%)	0.492
	No	249	101 (36.5%)	148 (33.9%)	
BMI Class	<25	484	253 (70.9%)	231(43.9%)	<0.001
	≥25	399	104 (29.1%)	295(56.1%)	
Fried criteria	0	256	128 (35.9%)	128 (24.3%)	<0.001
	1	190	69 (19.3%)	121 (23.0%)	
	2	126	38 (10.6%)	88 (16.7%)	
	3	145	51 (14.3%)	94 (17.9%)	
	4	92	33 (9.2%)	59 (11.2%)	
	5	74	38 (10.6%)	36 (6.8%)	
Frailty status	Healthy	256	128 (35.9%)	128 (24.3%)	<0.001
	Pre-frail	316	107 (30%)	209 (39.7%)	
	Frail	311	122 (34.2%)	189 (35.9%)	
ADL	Independent	522	208 (58.3%)	314 (59.7%)	0.490
	Semi-dependent	319	135 (37.8%)	184 (35%)	
	Dependent	42	14 (3.9%)	28 (5.3%)	
IADL	Functional independence	268	132 (61.4%)	136 (49.6%)	0.009
	Functional dependence	221	83 (38.6%)	138 (50.4%)	
Chronic disease	Yes	713	277 (77.6%)	436 (82.9%)	0.050
	No	170	80 (22.4%)	90 (17.1%)	
Diabetes	No	575	242 (67.8%)	333 (63.3%)	0.171
	Yes	308	115 (32.2%)	193 (36.7%)	
Hypertension	No	418	178 (49.9%)	240(45.6%)	0.216
	Yes	465	179 (50.1%)	286 (54.4%)	
Cardiovascular disease	No	748	311 (87.1%)	437 (83.1%)	0.102
	Yes	135	46 (12.9%)	89 (16.9%)	
Kidney disease	No	858	345 (96.6%)	513 (97.5%)	0.434
	Yes	25	12 (3.4%)	13 (2.5%)	
Articular disease	No	742	317 (88.8%)	425 (80.8%)	0.001
	Yes	141	40 (11.2%)	101 (19.2%)	
Pulmonary disease	No	841	331 (92.7%)	510 (97%)	0.004
	Yes	42	26 (7.3%)	16 (3%)	
Neurological disease	No	873	353 (98.9%)	520 (98.9%)	0.978
	Yes	10	4 (1.1%)	6 (1.1%)	
Digestive disease	No	854	343 (96.1%)	511 (97.1%)	0.381
	Yes	29	14 (3.9%)	15 (2.9%)	
Thyroid disease	No	867	357 (100%)	510 (97%)	0.001
	Yes	16	0 (0.0%)	16 (3%)	
Cancer	No	873	354 (99.2%)	519 (98.7%)	0.499
	Yes	10	3 (0.8%)	7 (1.3%)	

p: p-value (probability indicating the significance of the test); ADL: activities of daily living; IADL: instrumental activities of daily living

### Main results

**Prevalence of frailty:** according to Fried's frailty phenotype, 35.2% of participants could be considered frail, with a significant difference between sexes (35.9% of women vs. 34.2% of men, p <0.01). Pre-frail individuals represented 35.8%, with a predominance among women (39.7%) compared to men (30%), p <0.01, while robust participants constituted 29%, more frequent among men (35.9%) than among women (24.3%), p<0.01 (Table 2).

**Table 2:** comparison of sociodemographic characteristics of healthy, pre-frailty and frailty subjects

Variables		Total (883)	Frailty status			t/X2	P
			Healthy (256)	Pre-frail (316)	Frail (311)		
Age, years, mean (SD)		68.8±7.78	64.27±4.84	67.37±6.13	73.97±8.31	149.546^a^	<0.001
Gender	Male (%)	357(40.4)	128(35.9)	107(30)	122(34.2)	15.584^b^	<0.001
	Female (%)	526(59.6)	128(24.3)	209(39.7)	189(35.9)		
Age class	60-69(%)	522(59.1)	219(42)	215(41.2)	88(16.8)	238.532^b^	<0.001
	70-79(%)	247(28)	33(13.4)	85(34.4)	129(52.2)		
	≥80(%)	114(12.9)	4(3.5)	16(14)	94(82.5)		
Residence area	Urban (%)	242(27.4)	93(38.4)	75(31)	74(30.6)	14.424^b^	0.001
	Rural (%)	641(72.6)	163(25.4)	241(37.6)	237(37)		
Marital status	Single (%)	20(2.3)	8(40)	6(30)	6(30)	138.960^b^	<0.001
	Married (%)	509(57.6)	200(39.3)	200(39.3)	109(21.4)		
	Divorced (%)	42(4.8)	11(26.2)	20(47.6)	11(26.2)		
	Widowed (%)	312(35.3)	37(11.9)	90(28.8)	185(59.3)		
Education level	No schooling (%)	710(80.4)	176(24.8)	251(35.4)	283(39.9)	49.538^b^	<0.001
	Primary (%)	113(12.8)	46(40.7)	47(41.6)	20(17.7)		
	Secondary (%)	60(6.8)	34(56.7)	18(30)	8(13.3)		
Living arrangement	Alone (%)	137(15.5)	29(21.2)	44(32.1)	64(46.7)	10.118^b^	0.006
	With family (%)	746(84.5)	227(30.4)	272(36.5)	247(33.1)		

a : t value of ANOVA; ^b^: X2 value of chi-square test

**Associations between frailty, socio-demographic, and health profile of the elderly people:** our study shows that frailty is significantly higher in rural areas, with a rate of 37% compared to 30.6% in urban areas, p=0.001. Frailty is also more common among widowed participants, with a percentage of 59.3%, p<0.01. Similarly, it is more pronounced among participants with no formal education, displaying a rate of 39.9%, p <0.01. Furthermore, it is associated with the presence of chronic diseases as well as a BMI <25 (Table 2, Table 3).

**Table 3:** comparison of health characteristics of healthy, pre-frailty, and frailty subjects

Variables		Total/mean	Frailty status			t/X2	p
			Healthy	Pre-frail	Frail		
BMI		24.78±4.91	25.22±3.91	25.73±4.95	23.46±5.30	16.708^a^	<0.001
BMI Class	<25	484(54.8)	13127.1)	150(31)	208(41.9)	21.99^b^	<0.001
	≥25	399(45.2)	125(31.1)	166(41.6)	108(27.1)		
Subjective health	Good	232(26.3)	121(52.2)	87(37.5)	24(10.3)	172.825^b^	<0.001
	Average	228(25.8)	74(32.5)	85(37.3)	69(30.3)		
	Bad	349(39.5)	59(16.9)	128(36.7)	162(46.4)		
	Very bad	74(8.4)	2(2.7)	16(21.6)	56(75.7)		
Chronic disease	No	170(19.3)	91(53.5%)	46(27.1)	33(19.4)	63.143^b^	<0.001
	Yes	713(80.7)	165(23.1)	270(37.9)	278(39)		
Diabetes	No	575(65.1)	188(32.7)	193(33.6)	194(33.7)	11.101^b^	0.004
	Yes	308(34.9)	68(22.1)	123(39.9)	117(38)		
Cardiovascular disease	No	748(84.7)	225(30.1)	273(36.5)	250(33.4)	7.180^b^	0.028
	Yes	135(15.3)	31(23)	43(31.9)	61(45.2)		
Hypertension	No	418(47.3)	164(39.2)	133(31.8)	121(28.9)	41.085^b^	<0.001
	Yes	465(52.7)	92(19.8)	183(39.4)	190(40.9)		
Cancer	No	873(98.9)	255(29.2)	315(36.1)	303(34.7)	8.896^b^	0.012
	Yes	10(1.1)	1(10)	1(10)	8(80)		
Thyroid disease	No	867(98.2)	255(29.4)	310(35.8)	302(34.8)	4.966^b^	0.083
	Yes	16(1.8)	1(6.3)	6(37.5)	9(56.3)		
Digestive disease	No	854(96.7)	253(29.6)	306(35.8)	295(34.5)	6.999^b^	0.03
	Yes	29(3.3)	3(10.3)	10(34.5)	16(55.2)		
Neurological disease	No	873(98.9)	255(29.2)	315(36.1)	303(34.7)	8.896^b^	0.012
	Yes	10(1.1)	1(10)	1(10)	8(80)		
Pulmonary disease	No	841(95.2)	253(30.1)	306(36.4)	282(33.5)	23.355^b^	<0.001
	Yes	42(4.8)	3(7.1)	10(23.8)	29(69)		
Articular disease	No	742(84)	232(31.3)	273(36.8)	237(31.9)	23.801^b^	<0.001
	Yes	141(16)	24(17)	43(30.5)	74(52.5)		
Kidney disease	No	858(97.2)	255(29.7)	310(36.1)	293(34.1)	16.423^b^	<0.001
	Yes	25(2.8)	1(4)	6(24)	18(72)		

a : t value of ANOVA; ^b^: X^2^ value of chi-square test

**Factors associated with frailty:** the analysis revealed a significant association between frailty according to Fried's phenotype and various factors. In particular, advanced age (OR=1.16 (1.09-1.23); p <0.01), rural residence (OR=2.52 (1.35-4.71); p=0.004), subjective health (OR=2.35 (1.32-4.17); p=0.003), Activities of Daily Living (ADL) (OR=5.65 (2.33-13.69); p<0.001), and Instrumental Activities of Daily Living (IADL) (OR=3 (1.48-6.06); p=0.002) were identified as factors associated with frailty (Table 4).

**Table 4:** risk factor analysis for pre-frailty and frailty vs healthy

Variables	B	Wald	df	P	OR	95% CI for OR	
						Lower	Upper
Age (continuous)	0.153	26.392	1	0.000	1.165	1.099	1.236
Residence area (urban/rural)	0.927	8.493	1	0.004	2.528	1.355	4.717
Subjective health (good/average/bad)	0.855	8.568	1	0.003	2.352	1.327	4.17
ADL (independent/dependent)	1.733	14.737	1	0.000	5.655	2.335	13.698
IADL (functional independence / functional dependence)	1.1	9.404	1	0.002	3.004	1.487	6.069
Constant	-10.757	29.472	1	0.000	0		

B: logistic regression coefficient; Wald: Wald test for the null hypothesis that the coefficient is zero; df: degrees of freedom; OR: odds ratio; 95% CI for OR: 95% confidence interval for the odds ratio; ADL: activities of daily living; IADL: instrumental activities of daily living

## Discussion

In our study of 883 participants aged 60 and over, we observed a predominance of females. Indeed, 59.6% of the sample (526 individuals) were women. This trend is consistent with global observations where women generally have greater longevity than men. Nationally, this observation is confirmed by data from the High Commission for Planning (HCP), which indicates that women aged 60 and over represent 9.9% of the population, compared to 9.5% for men, according to the latest General Population and Housing Census (RGPH) of 2014 [13].

Our results indicate that more than two-thirds of the elderly people are illiterate, representing 80.4% of the studied sample. This figure aligns with data from the RGPH in 2014, which also reported an illiteracy rate of 70.5%, as well as with data from the National Survey on Population and Family Health (ENPSF) of 2018, which indicated a rate of 71.6%. It is observed that illiteracy is more prevalent among women, with 86.7% of women being illiterate compared to 71.1% among men. These results are consistent with the data from the ENPSF in 2018, which showed similar percentages: 83.6% for women compared to 60.1% for men [14].

This work also underscores that among the elderly aged 60 and over, the majority (57.6%) are married, while 35.3% are widowed, 4.8% are divorced, and 2.3% are single. These findings are also consistent with the data from the ENPSF in 2018, where it is reported that 70.6% of elderly aged 60 and over are married, 25.3% are widowed, 2.5% are divorced, and 1.7% are single. Family status presents marked disparities according to gender. Among the elderly aged 60 and above, 73.7% of men are married, compared to only 46.8% of women. Additionally, among all elderly women surveyed in our sample, 43.9% are widowed, while 22.7% of men are widowed. Thus, the proportion of men aged 60 and above who are married is nearly double that of elderly women. It appears that a portion of men aged 60 and over are likely remarried. However, it remains uncertain whether these remarriages occurred after or before the age of 60, and whether they resulted from widowhood or divorce [14].

In our study, the majority of participants, 84.5%, live with their families, while 15.5% live alone. This highlights the strong familial cohesion persisting in Morocco, despite the breakdown of the traditional family structure and the establishment of nuclear families. These findings align with those from the National Survey on Population and Family Health of 2018, which reported a percentage of 93.8% for elderly individuals living with their families and 6.2% living alone. The majority of participants in our sample have at least one chronic illness, with a percentage of 80.7%, surpassing the ENPSF 2018 data which indicated a rate of 64.4%. This finding suggests an increasing elderly population in Morocco. As the population ages, illnesses associated with longevity become more prevalent, posing a growing challenge for the Moroccan healthcare system, which is not yet fully equipped to handle elderly care.

Knowing the prevalence of frailty and its associated factors is crucial due to the rapid growth of the elderly population in Morocco. This study used the Fried physical phenotype to estimate frailty prevalence among a specific group of elderly people aged 60 and over. Results showed that 35.2% of the elderly were classified as frail. This rate is somewhat higher compared to studies using the Fried physical phenotype in other regions. For example, Santos-Eggimann *et al*. [15] reported a frailty prevalence of 17% among elderly aged 50 and older in 10 European countries, while Zhu *et al*. [16] found a frailty prevalence of 11.3% among those aged 70 to 84 years. In Canada, the frailty prevalence was 7.4% according to the Montreal Unmet Needs Study involving 740 elderly participants [17]. Additionally, another study in rural areas of China found a frailty prevalence of 6.8% among elderly people aged 60 and above [10]. The prevalence of frailty in the literature varies significantly, influenced by the assessment tools used and the characteristics of the studied population.

The prevalence we observed in our study is consistent with data in the literature on frailty and is comparable to that reported in a study conducted in France. In this study, a prevalence of 24.5% was observed in a population with an average age of 76, and 33.3% in people aged 75 and over, using the GFST (gérontopôle frailty screening tool) [18]. However, this is lower than the 59.8% prevalence found in a Korean study of people aged 65 and over [19]. The robust and pre-fragile participants in our study represent 29% and 35.8% respectively. While these percentages differ slightly from other studies, they reflect similar trends. For instance, a study conducted in Ankara reported rates of 42.5% and 44.1% [20]. While another study in Peru found percentages of 25.1% and 47.3% [21]. These findings broadly confirm the distribution of frailty states observed in different regions worldwide.

When comparing our results to studies from countries with similar sociodemographic and cultural contexts to Morocco, we find that many studies have reported much higher rates of frailty. For instance, in a study (2020) in Egypt, the prevalence of frailty was 33.5%, while other studies in the same country reported even higher figures, such as 64.7% according to Ali *et al*. [22]. However, a recent systematic review reported a frailty prevalence of 59% in Tunisia using the Fried frailty phenotype [23].

The results of our study reveal a higher prevalence of frailty among women (35.9% compared to 34.2% among men, p=0.000). This observation is consistent with the trend often observed in the literature, where frailty more frequently affects women according to several previous studies [10,17,24,25]. This predisposition among women can be attributed to their longer life expectancy compared to men. As age advances, the risk of frailty increases, thereby exposing women more to this risk. Furthermore, the association between frailty and female sex is documented in the literature, partly due to the higher proportion of women developing sarcopenia compared to men [26]. However, multivariate analyses reveal that sex is not significantly associated with frailty in our sample.

In our study, we observed an increasing prevalence of age-related frailty independent of gender, particularly in participants aged over 70, which corroborates previous work [20]. Advanced age was identified as the main risk factor for frailty in our cohort. In addition, unmarried elderly people with chronic diseases, a low level of education, and living in rural areas showed a notable prevalence of frailty, suggesting an increased risk in this population. These findings are consistent with those of other similar studies [26,27]. The increase in frailty in rural areas may be attributed to precarious living conditions, restricted access to healthcare services and possible delays in the diagnosis and treatment of chronic diseases, potentially accentuating the risk of frailty. In addition, our results highlighted a significant correlation between frailty and the presence of chronic illnesses, underlining the importance of taking these aspects into consideration for appropriate management.

Frailty is a complex multidimensional health condition involving various domains. Our study identified ADL, IADL, residential environment, and subjective health as factors significantly associated with frailty. This study is critically important for advancing research on elderly health in Morocco, serving as a foundational analysis of frailty within this population. By establishing initial data on frailty prevalence and risk factors, it is imperative to continue this work with further research to validate and expand upon our conclusions. Future studies will not only enhance our understanding of frailty in Morocco but also lay the groundwork for cross-cultural comparisons and targeted interventions aimed at improving the health and well-being of elderly people in the country.

**Limitations:** the main limitations of this study include the non-representative sample of the overall Moroccan population and the geographically restricted sample, which limits the generalizability of the results.

## Conclusion

This study revealed a notable prevalence of frailty among a group of elderly people aged 60 and over in Morocco, reaching 35.2%. This prevalence is significantly influenced by various factors such as age, gender, residential setting, marital status, educational level, subjective health perception, presence of chronic diseases, BMI, as well as ADL and IADL. These findings underscore the necessity of developing targeted prevention and intervention strategies to improve the quality of life of elderly people by considering these determinants of frailty. It is essential to promote community support programs, public health initiatives, and tailored healthcare services to reduce the burden of frailty and foster an active and healthy aging process. In particular, the implementation of frailty screening programs is crucial to identify at-risk elderly people and intervene early.

### What is known about this topic

- Frailty in elderly people is a state that precedes functional dependency;
- The early detection of frailty is a key factor in delaying or preventing the onset of functional dependency, thus providing an opportunity to improve the health and well-being of elderly people.

### What this study adds

- The prevalence of frailty among elderly people in Morocco is very high;
- The residence area is a determinant of frailty;
- Age and ADL are key factors in frailty among elderly people.
